# Optimizing data integration in trials that use EHR data: lessons learned from a multi-center randomized clinical trial

**DOI:** 10.1186/s13063-023-07563-y

**Published:** 2023-09-01

**Authors:** Sudha R. Raman, Laura G. Qualls, Bradley G. Hammill, Adam J. Nelson, Ester Kim Nilles, Keith Marsolo, Emily C. O’Brien

**Affiliations:** 1 Department of Population Health Sciences, Duke University School of Medicine, Durham, NC USA; 2 Duke Clinical Research Institute, Durham, NC USA; 3 Monash Heart, Monash University, Melbourne, VIC Australia

**Keywords:** Research design, Electronic health records, Pragmatic clinical trial as topic, Data quality

## Abstract

**Background:**

Despite great promise, trials that ascertain patient clinical data from electronic health records (EHR), referred to here as “EHR-sourced” trials, are limited by uncertainty about how existing trial sites and infrastructure can be best used to operationalize study goals. Evidence is needed to support the practical use of EHRs in contemporary clinical trial settings.

**Main text:**

We describe a demonstration project that used EHR data to complement data collected for a contemporary multi-center pharmaceutical industry outcomes trial, and how a central coordinating center supported participating sites through the technical, governance, and operational aspects of this type of activity. We discuss operational considerations related to site selection, data extraction, site performance, and data transfer and quality review, and we outline challenges and lessons learned. We surveyed potential sites and used their responses to assess feasibility, determine the potential capabilities of sites and choose an appropriate data extraction strategy. We designed a flexible, multimodal approach for data extraction, enabling each site to either leverage an existing data source, create a new research datamart, or send all data to the central coordinating center to produce the requisite data elements. We evaluated site performance, as reflected by the speed of contracting and IRB approval, total patients enrolled, enrollment yield, data quality, and compared performance by data collection strategy.

**Conclusion:**

While broadening the type of sites able to participate in EHR-sourced trials may lead to greater generalizability and improved enrollment, sites with fewer technical resources may require additional support to participate. Central coordinating center support is essential to facilitate the execution of operational processes. Future work should focus on sharing lessons learned and creating reusable tools to facilitate participation of heterogeneous trial sites.

**Supplementary Information:**

The online version contains supplementary material available at 10.1186/s13063-023-07563-y.

## Background

Over the past decade, there has been a significant shift in approaches to evidence generation in clinical medicine. Recognizing the need to transform conventional randomized clinical trials (RCT) to produce evidence more quickly and efficiently, key stakeholders have begun to test pragmatic methods for addressing common clinical questions [1, 2]. Central to many pragmatic study designs is the use of electronic health records (EHR), now actively used by 96% of US health systems [3], to streamline laborious and costly clinical trial processes. EHRs may be used in a number of ways within a clinical trial, including participant recruitment and screening, embedding randomization, and ascertainment of baseline and follow-up data on interventions and endpoints [4–6].

For trials that use EHR to ascertain clinical study data, referred to here as “EHR-sourced” trials, part of the consideration about whether EHR data are fit-for-use in regulatory environments is the uncertainty about how to leverage existing trial sites’ infrastructure to operationalize study goals. In addition to study design questions, this uncertainty includes questions about the feasibility of methods for integrating EHR data into clinical trial data infrastructure, and how to facilitate the scaling of these practices beyond early adopters [7–9]. As this field rapidly accumulates experience, the operational aspects of integrating EHR data into a large multi-site trial continue to be challenging, and examples are needed to realize the benefits of the study of large real-world populations.

Towards this end, the VESALIUS-CV EHR Demonstration Project was an industry, regulator, and academic research organization collaboration led by the Duke Clinical Research Institute (DCRI) with two aims. The first aim was to develop empirically driven recommendations to support the evaluation of real-world data (RWD) within large trials [7], and the second aim sought to assess the relative accuracy of EHR data about baseline clinical characteristics and clinical events of interest during longitudinal trial follow-up for trial participants, building on previous work that compared EHR data to traditional trial data (i.e., CRF and CEC) for trial participants for use within large multi-center trials [10].

An additional aim was to understand operational considerations, with a particular interest in assessing organization or site-level characteristics that drive readiness to participate in EHR-sourced multi-center trials. We conducted this work within the context of a contemporary multi-center pharmaceutical industry outcomes trial that used traditional processes of study visits, baseline, and follow-up event capture, electronic CRFs for data collection, and event adjudication to study the effect of lowering low-density lipoprotein cholesterol (LDL-C) with evolocumab on major cardiovascular events in high-risk adults. (ClinicalTrials.gov Identifier: NCT03872401).

This demonstration project utilized a central coordinating center, consisting of project management, data management/programming, and informatics expertise, to support participating US sites in addressing the technical, governance, and operational aspects of EHR use. In this paper, we discuss operational considerations related to site selection, data extraction, site participation, data transfer, and quality review, and we outline challenges and lessons learned. Lastly, we offer recommendations for future work to support clinical trial infrastructure and sites adapt to the use of EHR data.

## Main text

### Site selection — initial site survey

In the traditional site selection process, trial coordinating centers often consult internal site lists that summarize information about site characteristics, including enrollment capabilities and patient volume, as well as benchmarks and performance from previous trials. In addition to these considerations, an EHR-sourced trial requires consideration of the availability of additional informatics/information technology (IT) resources, which are often not known by the typical site staff members that are responsible for prospective participant recruitment. In a site interest survey for a prior demonstration project (HARMONY Outcomes EHR study) [10], we found that site-level technical resources did not consistently match those reported on the survey; specifically, there was variable understanding at the site level of the processes and governance of their EHR environment, which led to a divergence between their perceived and actual capabilities. Therefore, in this study, we modified several questions to more directly assess available site-level resources (Supplementary material 1). The final survey included questions regarding the site’s experience with EHR research, and technical capabilities along with specific questions related to this project. The team developed and launched this survey and used the responses to generate a preliminary site list for recruitment. Survey data were used to assess site interest and capabilities for participating, and match sites to the most appropriate data collection strategy given local resources.

### Site selection — data extraction strategies

Following receipt of survey data, we worked with site staff to assess their ability to participate in one of three EHR data extraction strategies. These strategies were designed, in part, based on our experience with the HARMONY Outcomes EHR study [10]. In the HARMONY Outcomes EHR study, several sites had the ability to organize data into a datamart (clinically relevant data stored in a query-able format), but we found that this process was not feasible at sites with limited experience working with EHR data or those with few IT resources.

**Fig. 1:**
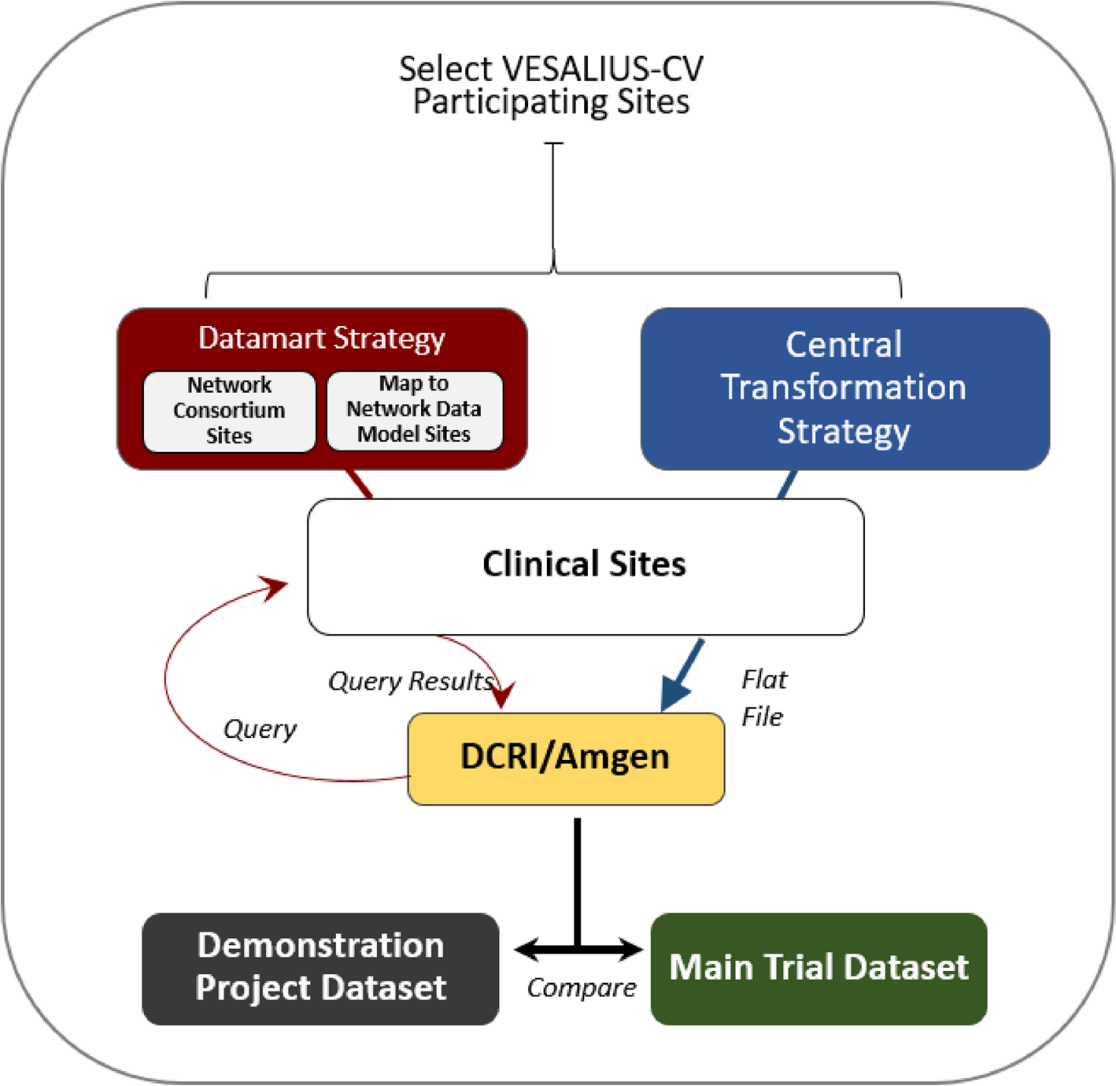
Data flow by data extraction strategy

To address the fact that only a minority of sites in this study had the requisite characteristics and resources to create a datamart, we designed a flexible, multimodal approach that enabled participation by sites with limited IT resources and/or technical capabilities while simultaneously leveraging site-level data that already exists in a query-able format (Fig. 1). The coordinating center provided data requirements (trial-specific list of the type and format of clinical concepts/variables) for each data strategy to the sites.

1. Network Consortium
  The Network Consortium strategy was offered to sites that were current participants in the Patient-Centered Clinical Research Network (PCORnet®) distributed research network who maintained an associated datamart. Sites choosing this option had already conducted the foundational work of mapping their EHR data to a common data model (CDM) and had made their data available in a query-able format. The CDM used for this study was a simplified version of the PCORnet Common Data Model [11]. The DCRI coordinating center provided data requirements to the sites to ensure that appropriate standardized data were already available in their datamart. Data requested from each site included demographic factors, medical and surgical history, selected lab results, and efficacy and safety endpoints aligned with endpoints from the main VESALIUS-CV trial dataset.
2. Map-to-CDM (Common Data Model)
  Sites that were not part of an existing Network Consortium but who wished to build a de novo research datamart to support the project objectives were offered the Map-to-CDM option. This option was offered to sites that previously participated in PCORnet or currently participate in another distributed research network. Although building a datamart entails substantial technical effort, this option was attractive to sites that might leverage the datamart for future research studies or that prefer to respond to a research query rather than create and share a full data extract as was required for the third strategy.
3. Central Transformation
  The Central Transformation strategy was created to support the inclusion of sites with more limited IT resources who were not part of a distributed research network or Network Consortium. Through this strategy, sites generated standardized reports for different EHR data domains (e.g., demographics, diagnoses, labs) for VESALIUS-CV trial patients who consented to participate in this study, which were then transferred to the coordinating center and transformed into a research datamart. Working with sites to obtain an extract of participant data may be less efficient than leveraging an existing research datamart, but shifting some technical work to a central team may expand both the types of participating sites (enhanced generalizability) and the number of patients included (statistical power).

Data extraction strategies were chosen in collaboration with site staff based on survey responses. Key qualifications for the Map-to-CDM or Network Consortium strategy included:

- Existence of an EHR platform at the enrolling site used for clinical care purposes, including inpatient hospitalizations;
- Access to an enterprise data warehouse or clinical data repository that stores structured data from their EHR system;
- Stated capability to extract EHR data from a data warehouse or repository according to standard specifications;
- Pre-existing institutional processes and policies for using EHR data for clinical research; and
- Demonstrated interest from the site-based clinical trial team and engagement of health system IT and analytical personnel at that site

**Fig. 2:**
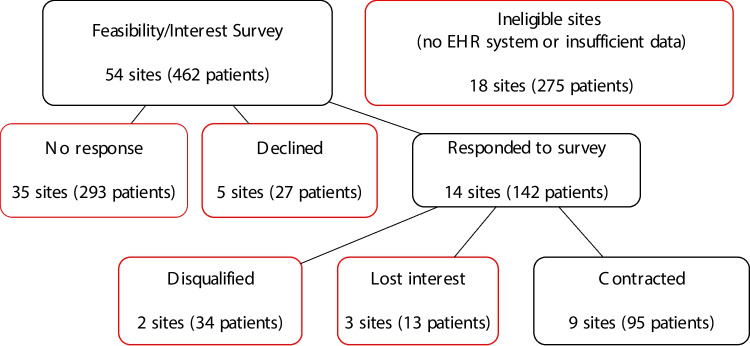
Flow diagram for VESALIUS-CV EHR study site participation

The project goal was to engage a total of 8–10 US sites, with representation across all 3 data collection strategies. Of the 54 US study sites, we determined that 18 had either insufficient data or did not have an EHR system. Of those who sent a feasibility survey (*n* = 54), 14 responded. As we discussed participation with each site, 5 sites either were found to be ineligible to participate or lost interest. Ultimately, we collaborated with 9 sites total, with 4 participating in the Central Transformation Strategy, 4 in the Network Consortium Strategy, and 1 in the Map-to-CDM strategy. Figure 2 displays the flow diagram of site selection, where the patient count is the number of patients participating in the VESALIUS-CV clinical trial as of January 7, 2022.

### Site performance

After the EHR data had been extracted and analyzed, we assessed a number of site participation measures and assessed their capabilities as shown in Table 1. Site participation measures (speed of contracting and IRB approval, total patients enrolled, and enrollment yield) were supplemented by a global assessment of site-specific capability within technical, operations, and regulatory domains based on our experience working with site staff over the course of the project (scaled 1–3, with 3 indicating the greatest site capability). Among the 9 sites with 95 trial patients, 89 patients were approached, and 75 patients consented to participate in the demonstration project. Of the 75 patients, 44 (58.7%) were from Central Transformation sites, 20 (26.7%) were from Network Consortium sites, and 11 (14.7%) were from the Map-to-CDM site. Contracting and IRB activities took 34 days longer on average at Network Consortium sites than at Central Transformation sites (average Network Consortium sites: 173, interquartile range (IQR) 167–222; average Central Transformation sites: 139, IQR 162–167). The contracting and IRB activity period for the single Map-to-CDM site was 60 days. Average enrollment yield was lower at Network Consortium sites (66.0%) than at Central Transformation sites (82.7%). As anticipated, technical capabilities were assessed as greatest at Network Consortium sites and less well-developed at Central Transformation sites. All sites had strong trial operations capabilities. In a post-hoc comparison of the sites’ demonstrated capability to their survey-reported capability, all but one site had concordance between reported and demonstrated capability (i.e., answers to all 14 survey questions matched their capabilities). One site overstated their technical capabilities and data availability in 3 of 14 survey responses.

**Table 1:** Site performance by data collection strategy

**Data collection strategy**	**Period from contracting/IRB initiation to completion (days)**	**# enrolled in trial**	**# approached for project**	**# consented**	**Enrollment yield** (consented/ enrolled)	**Capability rating**^**a**^		
						Technical	Trial operations	Regulatory/contracting
Central Transformation	164	5	5	4	80.0%	2	3	2
Central Transformation	160	27	25^b^	25^b^	92.6%	1	3	1
Central Transformation	177	12	9	7	58.3%	2	3	2
Central Transformation	56	8	8	8	100.0%	2	3	3
Map-to-CDM	60	12	12	11	91.7%	3	3	3
Network Consortium	119	1	1	1	100.0%	3	3	3
Network Consortium	118	3	3	3	100.0%	3	3	2
Network Consortium	241	2	1	0	0.0%	–	–	1
Network Consortium	215	25	25	16	64.0%	3	3	2
Total		95	89	75	78.9%			

^a^Global assessment ranging from 1–3, with 1 indicating the least capability and 3 indicating the greatest capability

^b^Demonstration study enrollment was capped at 25 participants

### Data transfer and quality review

For sites participating in the Map-to-CDM and Network Consortium strategies, the coordinating center was able to assess data quality before acquiring the study-specific data. First, for the Map-to-CDM sites, we distributed the existing PCORnet data curation code to query the content of tables formatted according to the CDM. The distributed code generated aggregate output tables that help determine whether the data conformed to specifications, maintained integrity across variables and across tables and trended as expected over time (see https://pcornet.org/data/). This first step was not necessary for the Network Consortium sites since they already do this as part of their participation in PCORnet. Next, the coordinating center developed and distributed a study-specific data characterization (SSDC) query to examine the availability of the diagnoses, procedures, and labs of interest, overall and by year. We found that all diagnoses, all procedures, and 6 of the labs of interest were captured by all sites for all years. Two labs of interest were not part of the standard clinical workflow at most institutions and therefore were not routinely available in the EHR data. This proactive data quality review and characterization process helped to ensure that the data met reasonable standards for data transformation, consistency, and quality.

The coordinating center then obtained the study-specific data for patients who consented to participate in the demonstration project. For sites participating in the Network Consortium or Map-to-CDM strategy, this was done using standard programs distributed to each study site for execution behind institutional firewalls. For sites participating in the Central Transformation strategy, the study utilized secure mechanisms by which sites sent data extracts of their EHR data in a flat file to the coordinating center for processing. Data received from Central Transformation sites represented all EHR data for the project participants. The coordinating center then transformed these data into the CDM format and performed the same study-specific data quality assessment that were done for the Network Consortium/Map-to-CDM sites. In their initial data submissions, all of the Central Transformation sites had some data quality issues, such as mapping issues and unexpected omissions. The coordinating center collaborated with the sites to help them rectify remediable issues (i.e., issues that were not a reflection of source data limitations). Following receipt and quality assessment of all site data, study data were aggregated and prepared for statistical analysis to support comparison to eCRF and CEC datasets from the trial*.* This work was done twice, first for an interim analysis (6 sites, 41 patients) and for a final analysis (8 sites, 75 patients).

### Lessons learned

The first lesson learned from this project is particularly striking — only 1 in 8 US sites participating in a large cardiovascular outcomes trial were interested in or able to participate in this effort to demonstrate how to ascertain data from EHR. Even when offered a more flexible set of options for data ascertainment, many interested sites were unable to participate in the demonstration project. A surprisingly high proportion (25%) did not have an EHR; among those with an EHR, many were either simply not interested (64.8%) or were non-responsive to the feasibility survey (9.3%), despite repeated email outreach to all institutions and telephone calls to the investigators at sites that were affiliated with a hospital system. The investigators at several sites were very interested in participating but were unable to get buy-in from the IT and/or contracting teams at their institutions. Engagement attempts were made in late 2019/early 2020 so response rates were likely affected by the strain of the COVID-19 pandemic. Although our study was a demonstration project and may not be fully representative of sites that may participate in future EHR-sourced trials, the widespread lack of site interest or ability to participate in EHR-based efforts has important implications for the scalability of such models. Given the substantial interest in using EHR within clinical trials across academic medical centers, governmental organizations, and industry sponsors, there is a need to further understand the landscape of site perspectives, capabilities, and performance, including site-level barriers to participation. Future EHR-sourced trials should consider the potential impact of EHR-related site selection on the generalizability of study findings, particularly if patient characteristics are substantially different from those of sites who are able to participate.

Second, our experiences with site selection and start-up paralleled those in many conventional clinical trials. One participating site did not enroll any patients in the demonstration project, in part due to contracting delays which led to few patient visits prior to the final query date. Similar to many prospective studies, there was variation in site performance with respect to study enrollment, with some sites enrolling much more effectively than others. In general, Central Transformation sites enrolled higher numbers of patients in the trial and had better demonstration project enrollment yield than Network Consortium sites. Given that the demonstration project largely overlapped with the first year of the COVID-19 pandemic, it is quite possible that enrollment in the trial and the demonstration project were paused due to the local diversion of research staff or the reallocation of resources. This problem may have disproportionally affected the Network Consortium sites, which are generally part of large academic health systems where the early COVID-19 burden was high.

Third, we observed important variations in regulatory and contracting capabilities by site. At one large academic center which used the Network Consortium strategy, EHR data are owned by the health system, but trial operations are generally executed by a separate entity. As a result, this site required separate contracts for these two entities, which contributed to a longer start-up process. Most Central Transformation sites used an external IRB, which may have supported more rapid completion of start-up activities, but this pattern was not universal; one Central Transformation site did not have its own IRB and did not have sufficient experience working with a central IRB for study oversight. This site agreed to rely on Duke’s IRB and a coordinating center team member drafted the application for submission and handled the IRB submissions. Given the novelty of the EHR-sourced approach for many trial sites, central availability of regulatory expertise and contracting resources are critical for ensuring that sites with varying levels of experience can be supported through the startup process.

Finally, even within the pre-defined data collection strategy types, we observed variations in the technical capabilities and ultimate approaches to data submission. The process for adapting the broad strategies at each site was iterative, with site-specific processes refined based on local data structure and central review of preliminary data submissions. All of the Central Transformation sites had to make corrections to their preliminary submissions. There were no resubmissions from the Network Consortium sites, likely due to our use of the same data domains previously used for other studies, which have each been through prior quality checks. At one Central Transformation site, the site did not receive an automated extract of inpatient data from the local hospital. As a result, site staff had to manually extract the data from the hospital EHR. From a coordinating center perspective, the quality control effort to evaluate data from this site was not substantially higher than for other Central Transformation sites; however, this may not be the case for trials with a large number of participants and/or many variables. Given the manual nature of the site-level work, however, the total effort required was much higher than for other Central Transformation sites. The technical lessons learned for Central Transformation sites are twofold: (1) providing central support to sites for data extraction was critical for enabling the capture of all key data elements; and (2) this support was continuous, relying on multiple rounds of review rather than a single orientation at study onset.

These lessons learned reflect a single trial experience, which may not be directly applicable to different types of trials or in settings/countries with heterogeneous data sources and trial processes. Additionally, though we included quantitative findings, any comparisons should be taken as preliminary and hypothesis generating. However, these observations are valuable when synthesized into the following recommendations.

### Recommendations for future work

We have several recommendations for future work within trial operations where EHR data is being used. First, it may be useful to consider a site “phenotype” that would reflect the variation in regulatory, contract, and technical capabilities at each site. This classification would support more efficient planning and allocation of coordinating center resources for support. Second, moving towards having EHR systems with capabilities to extract basic trial-relevant data about trial participants would enable more site participation with less reliance on local expertise. Third, a summary of site-specific resources that site staff may consult during the onboarding process and technical work may reduce the level of coordinating center support required. Finally, it will be important to establish open venues to share operational lessons learned, to facilitate the education of new research teams and spur development of institutional resources to guide investigators in this work.

## Conclusions

Our work explored the realities of offering multiple mechanisms for sites to participate in EHR-sourced trials that may broaden the number and type of sites able to participate in EHR-sourced trials, leading to more participation by sites and potentially greater generalizability. Given the wide variation in site-level resources and experience, central coordinating center support is essential to facilitate the successful execution of operational processes. Future work should focus on sharing lessons learned and creating reusable tools to facilitate participation of heterogeneous trial sites.

### Supplementary Information

**Additional file 1.** VESALIUS-EHR Demonstration Project Feasibility Survey.

**Additional file 2. **VESALIUS-EHR Demonstration Project.

## Data Availability

All data generated or analyzed about the study processes are included in this published article and appendices.

## Abbreviations

CDM: Common data model
CEC: Clinical events classification
CRF: Case report form
DCRI: Duke Clinical Research Institute
EHR: Electronic health records
IT: Informatics/information technology
LDL-C: Low-density lipoprotein cholesterol
PCORnet: Patient-Centered Clinical Research Network
RCT: Randomized clinical trials
RWD: Real-world data
SSDC: Study-specific data characterization
