# Supplementary material for: Optimizing data integration in trials that use EHR data: lessons learned from a multi-center randomized clinical trial
Source: Trials. 2023 Sep 1;24:566. doi: 10.1186/s13063-023-07563-y (PMC10474626; doi:10.1186/s13063-023-07563-y)
Supplement: Supplementary file 1 — Additional file 1. VESALIUS-EHR Demonstration Project Feasibility Survey. [file 13063_2023_7563_MOESM1_ESM.docx]

:

**Survey Participant Information**

Name(s) and role(s) of person(s) completing the survey:

Institution:

Email:

1. **Has your institution ever participated in a research study where EHR data were extracted through an automated process (i.e., not through chart abstraction)?**

Yes

No

Unsure

1. **Which Electronic Health Record system(s) do you use (check all that apply)? If you know the version, please provide it.**

AllScripts (version: _________________)

AthenaHealth (version: ______________)

Cerner (version: ______________)

eClinicalWorks (version: ______________)

Epic (version: ______________)

NextGen (version: ______________)

McKesson (version: ______________)

PracticeFusion (version: ______________)

Other (please specify)

1. **Does your institution plan to transition to a new EHR vendor/platform in 2020 or 2021?**

Yes

No

Unsure

1. **Where is your EHR data stored?**

Locally

In a cloud-based EHR system

Unsure

1. **Does your institution store its data in a data warehouse, data lake or other centralized resource?**

Yes

No

Unsure

1. **Has your EHR data been mapped to any of the following common data models (check all that apply)?**

PCORnet

Sentinel

OMOP

i2b2

FHIR

Other (please specify)

1. **Are there any restrictions on the patient populations included in your EHR system?**

Yes

Unsure

No (please describe)

1. **Please indicate which of the following data are included in your EHR system or data warehouse**

Date of Birth:  Yes  No  Unsure

Sex:  Yes  No  Unsure

Race:  Yes  No  Unsure

Ethnicity:  Yes  No  Unsure

Diagnoses:  Yes  No  Unsure

Procedures:  Yes  No  Unsure

Serum creatinine (quantitative results):  Yes  No  Unsure

Triglycerides (quantitative results):  Yes  No  Unsure

Cholesterol (quantitative results):  Yes  No  Unsure

Lipoprotein-A (quantitative results):  Yes  No  Unsure

1. **Please indicate which care settings are included in your EHR system (check all that apply)**

Inpatient hospitalizations

Emergency room visits

Physician office visits

Outpatient hospital visits

1. **Does the availability of diagnosis, procedures or lab results differ by care setting?**

No, it does not vary

Unsure

Yes, it does vary (please describe any differences):

1. **If diagnoses are available in your EHR and/or data warehouse, please indicate how they are recorded (check all that apply)**

Captured in an unstructured format (e.g. notes)

Captured in a structured format using ICD codes

Captured in a structured format using SNOMED codes

Captured in a structured format using other coding terminologies (e.g., IMO, local codes)

Unsure

1. **If procedures are available in your EHR and/or data warehouse, please indicate how they are recorded (check all that apply)**

Captured in an unstructured format (e.g. notes)

Captured in a structured format using ICD codes

Captured in a structured format using CPT codes

Captured in a structured format using other coding terminologies (e.g., local codes)

Unsure

1. **If laboratory test results are available in your EHR and/or data warehouse, please indicate how they are recorded (check all that apply)**

Captured in an unstructured format (e.g. notes, HL7 feeds)

Captured in a structured format using LOINC codes

Captured in a structured format using other coding terminologies (e.g., local codes)

Unsure

1. **Which sources of death information are captured in your EHR and/or data warehouse (check all that apply)?**

In-hospital death

Family report

National Death Index (NDI)

Other (please describe)

Unsure

Thank you for your interest in the Vesalius-EHR Demonstration Project. Please return your completed survey to [laura.qualls@duke.edu](mailto:laura.qualls@duke.edu).
