# Supplementary material for: Optimizing data integration in trials that use EHR data: lessons learned from a multi-center randomized clinical trial
Source: Trials. 2023 Sep 1;24:566. doi: 10.1186/s13063-023-07563-y (PMC10474626; doi:10.1186/s13063-023-07563-y)
Supplement: Supplementary file 2 — Additional file 2. VESALIUS-EHR Demonstration Project. [file 13063_2023_7563_MOESM2_ESM.docx]

**Variables of interest**

*Demographics*

- Age at enrollment
- Sex
- Race
- Hispanic ethnicity

*Medical history*

- Coronary artery disease
- Cerebrovascular disease
- Diabetes Mellitus
- Peripheral artery disease
- Myocardial infarction
- Stroke
- Coronary artery bypass grafting (CABG)
- Percutaneous coronary intervention (PCI)
- Heart failure
- Hypertension
- Atrial fibrillation

*Local Laboratory Values*

- Serum creatinine
- LDL cholesterol
- Total cholesterol
- Non-HDL-cholesterol
- Fasting triglycerides
- Lipoprotein A (Lp(a)) above or below 125 nmol/L

*Outcomes*

- Myocardial infarction
- Ischemic stroke
- Hemorrhagic stroke
- Stroke, other/unspecified
- Coronary revascularization procedure (CABG/PCI)
- Peripheral arterial revascularization procedure
- All-cause mortality
- Heart failure
